# Clinical effectiveness and safety of self-expandable implantable bulking agents for faecal incontinence: a systematic review

**DOI:** 10.1186/s12876-022-02441-4

**Published:** 2022-08-17

**Authors:** Lucia Gassner, Claudia Wild, Melanie Walter

**Affiliations:** 1 HTA Austria - Austrian Institute for Health Technology Assessment GmbH, Garnisongasse 7/20, 1090 Vienna, Austria; 2 University of Vienna, Vienna, Austria

**Keywords:** Faecal incontinence, Bulking agents, Gatekeeper™, Sphinkeeper™, Systematic review

## Abstract

**Purpose:**

The purpose of this systematic review is to evaluate whether self-expandable implantable vs non-self-expandable injectable bulking agents (second-line therapies) are equal/superior in terms of effectiveness (severity, quality of life [QoL]) and safety (adverse events) for faecal incontinence (FI).

**Methods:**

A systematic review was conducted, and five databases were searched (Medline via Ovid, Embase, Cochrane Library, University of York Centre for Reviews and Dissemination, and International Network of Agencies for Health Technology database). In-/exclusion criteria were predefined according to the PICOS scheme. The Institute of Health Economics risk of bias (RoB) tool assessed studies' internal validity. According to the Grading of Recommendations, Assessment, Development and Evaluation approach, the strength of evidence for safety outcomes was rated. A qualitative synthesis of the evidence was used to analyse the data.

**Results:**

The evidence consists of eight prospective single-arm, before-after studies (166 patients) fulfilling the inclusion criteria for assessing clinical effectiveness and safety of implantable bulking agents. FI severity statistically significantly improved in five of seven studies rated by the Cleveland Clinic FI Score and in three of five studies measured by the Vaizey score. Statistically significant improved disease-related QoL was found in one of five studies measured by the FI QoL Score and in one of two studies rated by the American Medical Systems score. Procedure-related adverse events occurred in 16 of 166 patients (i.e., intraoperative complications, anal discomfort and pain). Device-related adverse events occurred in 48 of 166 patients, including prostheses’ dislodgement and removed/extruded prostheses. Studies were judged with moderate/high RoB. The strength of evidence for safety was judged to be very low.

**Conclusion:**

Implantable bulking agents might be an effective and safe minimally invasive option in FI treatment if conservative therapies fail. FI severity significantly improved, however, effects on QoL need to be explored in further studies. Due to the uncontrolled nature of the case series, comparative studies need to be awaited.

**Supplementary Information:**

The online version contains supplementary material available at 10.1186/s12876-022-02441-4.

## Introduction

Faecal incontinence (FI), a highly prevalent condition, is the involuntary loss of intestinal contents due to an impaired ability to control the release of faeces/flatus [1–3]. Patients with FI suffer from a complex health problem causing considerable physical and social impairments leading to massive limitations in the quality of life (QoL) due to isolation, shame, and social rejection [4]. These stigmatising conditions adversely affect psychological well-being [3, 5–7]. The prevalence is estimated to 2–20% in the adult population and increases with age [6, 7]. The true number of patients is unknown because FI is still a taboo subject [8].

Functional and/or structural abnormalities of the external anal sphincter (EAS) and internal anal sphincter (IAS) are more frequent in women, caused mainly by obstetric traumas [5]. Commonly, FI is caused by a weak sphincter muscle and/or pelvic floor muscle [4]. The choice of appropriate treatments can be challenging due to the multifactorial aetiology, pathophysiological mechanisms, and difficulty in accurately defining the cause [2, 6, 9, 10].

The majority of FI patients profit from conservative measures (e.g., pelvic floor muscle, biofeedback training). In a retrospective clinical review [11] with 574 FI patients, only 9% required surgical interventions, and the importance of conservative measures in FI has to be highlighted. If conservative therapies fail, alternatives such as bulking agents are second-line options [12], preventing further declines or improving FI symptoms [13]. The International Consultation on Incontinence, an expert panel of incontinence specialists, proposed a treatment algorithm. It recommends a stepwise approach to FI treatments, i.e., surgical measures (e.g., colostomy, sacral nerve stimulation, sphincteroplasty, artificial sphincter) *only* if conservative treatments have failed [14, 15].

Injectable and implantable bulking agents can be considered as a minimally invasive option in FI management [16]. *Injectable* bulking agents, i.e. non-self-expandable prostheses, such as Solesta®, Bulkamid™, PTQ™, Durasphere®, and Permacol™, are injected around or into the anal canal [3, 4]. The main clinical indication for injectable bulking agents is IAS disruption/dysfunction, causing passive FI [3]. Bulking agents' injections vary depending on the clinical indication and type of substance used [3].

*Implantable* bulking agents, i.e. self-expandable prostheses, are thin cylinders becoming thicker, shorter and softer 48 hours after insertion due to their hydrophilic properties, expecting to improve FI [3, 4]. Implantable bulking agents can be seen as the latest anal bulking agents, available as Gatekeeper™ and Sphinkeeper™ devices [3, 4]. Gatekeeper™ prostheses consist of four to six self-expandable, solid, thin cylinders [3, 5]. Sphinkeeper™ can be seen as the advancement of Gatekeeper™ due to its higher number of implanted prostheses (10 prostheses) [16, 17]. TÜV Rheinland Italia S.r.l approves both Gatekeeper™ and Sphinkeeper™, which hold a CE mark (CE certificate number HD60147418), first registered in 2010 for the indication of FI [18].

**Fig. 1:**
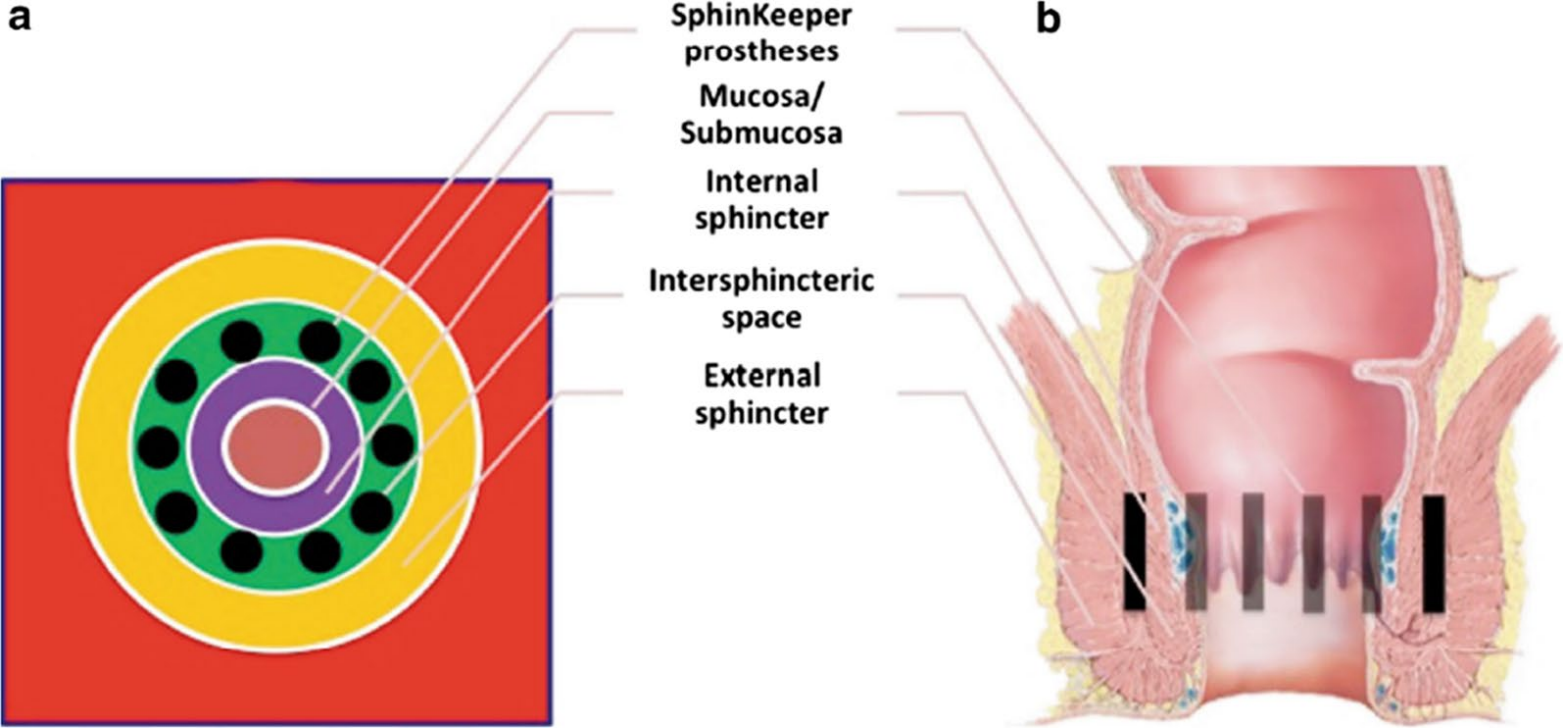
The site of Sphinkeeper™ implantation within the interspincteric space. It shows the ten prostheses around the entire circumference of the internal anal sphincter: transverse plane (panel **A**) and frontal plane (panel **B**)

The implantation technique is conducted as a day case [3]. After an incision is made, the prosthesis is released into the intersphincteric space of the anal canal utilising a custom-made gun [3, 5] (Fig. 1). The procedure is relatively simple to perform from a technical perspective, but prostheses' placement and deployment can occur [1]. Implanting prostheses into the intersphincteric space shall avoid migration/extrusion [3]. Prostheses are not expected to move due to their rapid increase in volume, embedded within the intersphincteric space pushing the IAS inwards and the EAS outwards [3].

This systematic review aims to evaluate whether self-expandable implantable bulking agents are superior or equal to non-self-expandable injectable bulking agents as second-line therapy in terms of clinical effectiveness and safety for FI after the failure of conservative interventions (first-line therapy). It is commissioned by the Austrian Federal Ministry of Social Affairs, Health, Care and Consumer Protection and provides decision support for reimbursement and implantable bulking agents' inclusion in the Austrian hospital benefit catalogue.

## Methods

A systematic review of clinical effectiveness and safety of self-expandable implantable compared to non-self-expandable injectable bulking agents was conducted, applying the European Network of Health Technology Assessment (EUnetHTA) Core Model for rapid relative effectiveness assessment [19, 20]. The Preferred Reporting Items for Systematic Reviews and Meta-Analyses (PRISMA) guidelines were used as reporting standards [21, 22]. This article at hand is an output of an HTA report [23], which is an update of a previous HTA report in 2015 (decision support document Nr 87 [4]). Therefore, the outcomes (e.g., tools measuring FI severity [Wexner Cleveland Clinic Faecal Incontinence Score, CCFIS; Vaizey score] and QoL [Faecal Incontinence Quality of Life Scale, FIQL; American Medical Systems score, AMS) were predefined and derived from the previous report. The AMS score is a modification of the FIQL [24].

### Literature search and eligibility criteria

The systematic literature search (see Additional file 1) was carried out on 17/12/2020 (update search 30/05/2022) in Medline via Ovid, Embase, the Cochrane Library, the University of York Centre for Reviews and Dissemination, and the International Network of Agencies for Health Technology database. The search was limited to 2015 to 2020 and articles published in English or German. Study designs for clinical effectiveness and safety were limited to randomised controlled trials, prospective non-randomised controlled trials, and prospective single-arm, before-after studies. Only adults with FI in who conservative treatment interventions failed were included. Implantable were compared to injectable bulking agents in terms of clinical effectiveness and safety outcomes. According to the PICOS scheme [25] (i.e., Population, Intervention, Comparison, Outcome, and Study design), the eligibility criteria for relevant studies are summarised in Table 1. Furthermore, a search in three clinical trials registries (ClinicalTrials.gov, WHO International Clinical Trials Registry Platform, EU ClinicalTrials [European Union Drug Regulating Authorities Clinical Trials Database]) was conducted on the 14/01/2021 (update search 31/05/2022) to identify ongoing and unpublished studies.

**Table 1:** Inclusion criteria based on the PICOS (Population, Intervention, Control, Outcomes, Study design) tool [25]: clinical effectiveness and safety for implantable bulking agents for faecal incontinence

Population	Adult patients (≥ 18 yrs) with faecal incontinence (FI) in who conservative treatment interventions failed ICD-10 codes: Faecal incontinence (R15), Other specified diseases of anus and rectum (K62.8)
Intervention	Bulking agents—self-expandable **implantations** (= products Gatekeeper™ and Sphinkeeper™) as second-line therapy
Control	Bulking agents—non-self-expandable **injections**
Outcomes	
Clinical effectiveness	FI severity (Scores: Wexner Cleveland Clinic Faecal Incontinence Score [CCFIS], Vaizey score) Disease-related quality of life (Scores: Faecal Incontinence Quality of Life Scale [FIQL], American Medical Systems score [AMS]) Sustainability of interventions: Durability of effectiveness > 6 months
Safety	Procedure-related adverse events Device-related adverse events
Study design	Randomised controlled trials Prospective non-randomised controlled trials Prospective uncontrolled trials
Publication period	2015–2020
Languages	English, German

### Study selection

**Fig. 2:**
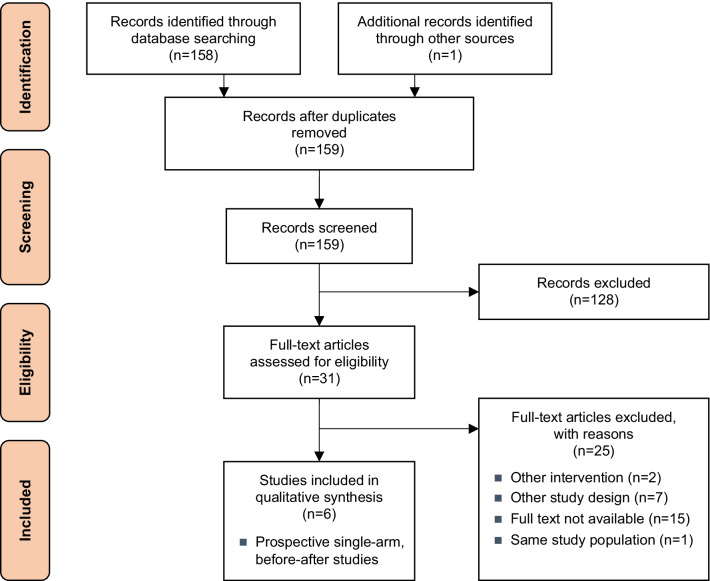
PRISMA (Preferred Reporting Items for Systematic Reviews and Meta-Analyses) flow chart of study selection [21, 22] (update search not presented in PRISMA)

The selection process is displayed in Fig. 2. The systematic literature search resulted in 158 hits after deduplication. The manufacturer (THD s.p.A.) of the assessed products (Gatekeeper™, Sphinkeeper™) submitted one additional publication, an accepted but still unpublished paper [26], resulting in overall 159 hits. By hand-search, no additional studies were found.

Abstracts were screened, and potentially relevant full-text articles were reviewed by two independent researchers (LG, CW) based on the predefined inclusion criteria. In case of disagreement, a third researcher (MW) was involved in solving differences.

### Selected outcomes

Within the scoping phase, the following patient-relevant effectiveness and safety outcomes were chosen as critical following a Cochrane review [27]: FI severity, disease-related QoL, and procedure and device-related adverse events.

#### Outcomes clinical effectiveness

*FI severity* was measured by two validated scoring systems [24, 28–30], assessing FI severity and documenting/evaluating treatment outcomes: The *CCFIS* [30] and the *Vaizey* score [31]. Clinical improvement of the CCFIS denotes a minimum of 50% reduction in the scale score relative to the preoperative score [12]. *Disease-related QoL* was measured by two validated scoring systems [24, 28–30]: The *FIQL* [24, 32] and the *AMS* [24].

#### Outcomes safety

Safety outcomes were selected in terms of *procedure-related adverse events* (i.e., intraoperative complications; postoperative complications, morbidity; infection, sepsis, inflammation; anal discomfort, pain, analgesia > 48 h; adverse effect, complication, reaction), and *device-related adverse events* (i.e., prostheses' dislodgement, removed/extruded prosthesis) [4].

### Data extraction

Single data extraction method was used by one author (LG), validated by a second reviewer (CW). The extraction tables (Tables 2, 3) were completed with variables according to the PICOS schema [25]. Effect measures were not reported in the included studies.

**Table 2:** Data extraction table: clinical effectiveness and safety of implantable bulking agents (Gatekeeper™ and Sphinkeeper™ [THD s.p.A., Italy]) for faecal incontinence

Product	Gatekeeper™				Sphinkeeper™		
References	Brusciano [35]	De la Portilla [12]	Ratto [10]		Litta [26]	La Torre [7]	Ratto [17]
Country	Italy	Spain	Italy		Italy	Italy	Italy
Sponsor	None	NR	NR		None, (1 CoI)	NR	NR (no CoI)
Comparator	None	None	None		None	None	None
Study design	Prospective, before-after, single-arm, single-centre	Prospective, before-after, single-arm, single-centre	Prospective, before-after, single-arm, multi-centre		Prospective, before-after, single-arm, single-centre	Prospective, before-after, single-arm, single-centre	Prospective, before-after, single-arm, single-centre feasibility study
Conducted in	01/2014–04/2016	NR	06/2011–12/2013		03/2016–10/2018	12/2016–02/2018	07/2014–04/2015
Indication	Passive FI	Passive FI	FI not specified (passive, urge, or mixed)		FI not specified (passive, urge, or mixed)	FI not specified (passive, urge, or mixed)	FI Passive (n = 4), urge (n = 4), mixed (n = 3)
Intervention	4 (n = 4) or 6 (n = 16) prostheses	6 prostheses	6 prostheses		10 prostheses	10 prostheses	10 prostheses
Number of pts at baseline	20 (20 females)	7 (6 females)	54 (37 females)^a^		45 pts	13 (10 females)	10 (5 females)
Number of pts analysed	20 females	7 (6 females)	54 (37 females)		39 (34 females)	13 (10 females)	10 (5 females)
Loss to FU, n (%)	0 (0)	0 (0)	0 (0)		3 (6.7) + 3 (6.7) excluded (unusable data)	0 (0)	0 (0)
Median age of patients, yrs median (range)	59 (24–77)	Mean age 55.6 (50.5–57.2)	66 (41–80)		68 (58–74)	NR (> 18)	70 (20–75)
Inclusion criteria	FI onset ≥ 6 months Symptoms being refractory to all standard conservative measures	Passive FI for mean duration of 6 ± 2 years IAS lesion extending < 60° of the anal circumference (mean 38 ± 4.0°)	18–80 years FI onset ≥ 6 months FI episodes > 1x/week Resistant to other conservative treatments Intact anal sphincters or lesion only of IAS maximum circumferential extension of 60°		> 18 yrs FI onset ≥ 6 months FI episodes > 1x/week Failure of conservative treatment IAS and/or EAS defects < 120° Consent to the study Attendance of all FU visits	> 18 yrs FI onset ≥ 6 months FI episodes > 1x/week Resistant to conservative treatments Intact anal sphincters or sphincter injury (IAS, EAS, or both) IAS and EAS defects	18–80 years FI onset ≥ 6 months FI episodes > 1x/week Willingness to perform FU
Exclusion criteria	IAS lesion > 60° and/or EAS lesion > 90° Presence of active perianal sepsis Severe anal scarring Active treatments for anal or rectal cancer IBD with anorectal involvement	NR	IAS lesion > 60° or EAS lesion Previous anal surgery for FI Active perianal sepsis Severe anal scarring IBD with anorectal involvement Anal or rectal cancer Uncontrolled endocrine, metabolic or neurological disease Congenital anorectal malformation		Diagnosis of cancer IBD Acute anorectal sepsis Refractory chronic diarrhoea Rectal bleeding Sphincter defects > 120°	Malignant neoplasms Rectal bleeding Congenital anorectal malformations IBD Sepsis Obstructive defaecation syndrome Neurological disease Coagulation disorders	Malignancies under treatment Rectal bleeding Chronic diarrhoea IBD Acute anorectal sepsis Concomitant rectal prolapse Obstructive defaecation syndrome Neurological disease Coagulation disorder
Clinical outcome measures	Clinical effectiveness: FI severity (CCFIS) Safety: NR	Clinical effectiveness: FI severity (CCFIS [= Wexner], diary), QoL (FIQL) Safety: NR	Clinical effectiveness: FI severity (CCFIS, Vaizey, diary), QoL (AMS, FIQL) Safety: NR		Clinical effectiveness: FI severity (CCFIS, Vaizey) Safety: NR	Clinical effectiveness: FI severity (CCFIS, diary), QoL (FIQL) Safety: NR	Clinical effectiveness: FI severity (CCFIS, Vaizey, diary), QoL (AMS, FIQL) Safety: specified parameters
FU, months	1, 3, 6, 12, 24, 36	1, 3, 12	1, 3, 12 (FU median 12 ± 4)		1, 3, 6, annually (FU median 14 months [IQR, 7–23])	6	3
Outcomes							
Clinical effectiveness							
Faecal incontinence severity							
CCFIS (mean ± SD [12, 35], median [range] [10, 17], median [1. and 3. quartile] [26] or mean [range] [7])							
			Group A Pts with ≥ 75% improvement in FI (n = 30)	Group B Pts with < 75% improvement in FI (n = 24)			
Preoperative	12.4 ± 1.8	16.0 ± 4.0	12 (3–20)		12 (9–15)	12.46 (10–15)	10 (5–17)
			13 (3–20)	9 (3–20)			
Postoperative 3 months	4.9 ± 1.5; *p* < 0.0001	10.4 ± 3.2; *p* < 0.01	4 (0–19); *p* < 0.001	6 (0–16); *p* = 0.002	NR	NA	NR
Postoperative 6 months	NR	NA	NA		NR	8.91 (6–12); *p* < 0.05	NA
Postoperative 12 months	NR	10.1 ± 3.1; *p* < 0.01	5 (0–16); *p* < 0.001		NR	NA	NA
			4 (0–22); *p* < 0.001	5 (1–16); *p* = 0.002^b^			
Postoperative 14 months (median)	NA	NA	NA		7 (5–11); *p* = 0.001	NA	NA
Postoperative 24 months	NR	NA	NA		NR	NA	NA
Postoperative 36 months	4.9 ± 1.7; *p* < 0.0001 (4.4 ± 1.0; *p* < 0.0001 with 6 prostheses)	NA	NA		NA	NA	NA
Vaizey (mean ± SD [12, 35], median [range] [10, 17], median [1. and 3. quartile] [26] or mean [range] [7])							
Preoperative	NA	NA	14 (3–24)		15 (13–18)	NA	13 (7–16)
			15 (3–24)	12 (5–21)			
Postoperative 3 months	NA	NA	4 (0–19); *p* < 0.001	8.5 (0–18); *p* = 0.012	NR	NA	NR
Postoperative 6 months	NA	NA	NA		NR	NA	NA
Postoperative 12 months	NA	NA	6.5 (0–17); *p* < 0.001		NR	NA	NA
			4 (0–22); *p* < 0.001	8 (2–17); *p* = 0.012			
Postoperative 14 months	NA	NA	NA		11 (7–14); *p* = 0.001	NA	NA
Disease-related QoL							
FIQL: Lifestyle (mean ± SD [12], median [range] [10, 17] or mean [range] [7])							
Preoperative	NA	NR	NR		NA	2.62 (2.2–3.1)	3.2 (2.1–3.8)
Postoperative 3 months	NA	NR; NS	NR		NA	NA	NR
Postoperative 6 months	NA	NA	NA		NA	3.2 (2.9–3.5); NS	NA
Postoperative 12 months	NA	NR; NS	NR; *p* = 0.01		NA	NA	NA
FIQL: Coping/behaviour (mean ± SD [12], median [range] [10, 17] or mean [range] [7])							
Preoperative	NA	NR	NR		NA	1.97 (1.7–2.2)	2.0 (1.2–2.9)
Postoperative 3 months	NA	NR; NS	NR		NA	NA	NR
Postoperative 6 months	NA	NA	NA		NA	2.37 (2–2.6); NS	NA
Postoperative 12 months	NA	NR; NS	NR; *p* = 0.001		NA	NA	NA
FIQL: Depression/self-perception (mean ± SD [12], median [range] [10, 17] or mean [range] [7])							
Preoperative	NA	NR	NR		NA	2.96 (2.7–3.2)	3.6 (2.1–3.9)
Postoperative 3 months	NA	NR; NS	NR		NA	NA	NR
Postoperative 6 months	NA	NA	NA		NA	3.39 (3.1–3.6); NS	NA
Postoperative 12 months	NA	NR; NS	NR; *p* = 0.029		NA	NA	NA
FIQL: Embarrassment (mean ± SD [12], median [range] [10, 17] or mean [range] [7])							
Preoperative	NA	NR	NR		NA	2.46 (2–2.8)	2.3 (2.0–4.0)
Postoperative 3 months	NA	NR; NS	NR		NA	NA	NR
Postoperative 6 months	NA	NA	NA		NA	3 (2.7–3.4); NS	NA
Postoperative 12 months	NA	NR; NS	NR; *p* = 0.001		NA	NA	NA
AMS (median [range] [10, 17] or mean [range] [7])							
Preoperative	NA	NA	87 (27–120)		NA	NA	80 (26–114)
			94 (28–120)	82 (27–120)			
Postoperative 3 months	NA	NA	32 (0–182); *p* < 0.001	38 (0–80); *p* < 0.001	NA	NA	NR
Postoperative 6 months	NA	NA	NA		NA	NA	NA
Postoperative 12 months	NA	NA	43.5 (0–106); *p* < 0.001^c^		NA	NA	NA
			32.5 (0–120); *p* < 0.001	59 (1–105); *p* < 0.001			
Outcomes							
Safety (n [%])							
Procedure-related adverse events							
Intraoperative complications	NR	0 (0)	3 (6) prostheses extruded during surgery		0 (0)	0 (0)	NR
Postoperative complications/morbidity	NR	0 (0)	0 (0)		0 (0)	0 (0)	0 (0)
Infection/sepsis/inflammation	NR	0 (0)	0 (0)		0 (0)	0 (0)	0 (0)
Anal discomfort/pain, analgesia > 48 h	NR	1 (14.3) for 4 days	7 (13) for 4.4 (3.8) days		2 (5.1)	0 (0)	1 (10); for 1 week after surgery
Adverse effect/reaction/general complication	0 (0)	NR	NR		0 (0)	NR	0 (0)
Device-related adverse events							
Dislodgement of prostheses	4 (20)	5 (71.4) 24/42 prostheses in 5/7 pts	3 (6)		18 (46.2)	1 (7.7)	0 (0)
Prosthesis removed/extruded	NR	1 (14.3)	NR		NR	2 (15.4)	0 (0)

*AMS* American Medical Systems score, *CCFIS* Cleveland Clinic Faecal Incontinence score, *CoI* conflict of interest, *EAS* external anal sphincter, *IBD* inflammatory bowel diseases, *FI* faecal incontinence, *FIQL* Faecal Incontinence Quality of Life score, *FU* follow-up, *IAS* internal anal sphincter, *IQR* interquartile range, *m* mean, *NA* not available, *n* number of patients, *NR* not reported, *NS* not significant, *p.m.* per month, *pts* patients, *p.w.* per week, *QoL* quality of life, *SD* standard deviation, *UK* United Kingdom, *Wexner* Wexner scale assessment, *yrs* years. A description of the scores can be found in the legend of Table 3

^a^Divided into two groups: Patients with ≥ 75% improvement in FI (group A; n = 30) and patients with < 75% improvement in FI (group B; n = 24)

^b^24 patients (44%) reported less than 75% improvement in faecal incontinence parameters at 1-year follow-up

^c^Discrepancy could be observed as Group B had a range from 1–105 (not 106)

**Table 3:** Data extraction table from update search: Clinical effectiveness and safety of implantable bulking agents (Sphinkeeper™ [THD s.p.A., Italy]) for faecal incontinence

Product	Sphinkeeper™	
References	Dawoud [36]	Colbran [37]
Country	Austria	Australia
Sponsor	None (no CoI)	None (no CoI)
Comparator	None	None
Study design	Prospective, before-after, single-arm, single-centre	Prospective, before-after, single-arm, single-centre
Conducted in	2018–2020	02/2018–09/2019
Indication	Refractory FI	FI not specified
Intervention	Median: 9 prostheses	10 prostheses
Number of pts at baseline	11 (9 females)	13 (11 females)
Number of pts analysed	11 (9 females)	12 (females: NR)
Loss to FU, n (%)	0 (0)	1 (7.7)
Age of patients, yrs median (range) [36] Mean age ± SD [37]	75 (46–89)	56.7 ± 12.7
Inclusion criteria	Failure to respond to conservative treatment	> 18 yrs FI symptoms > 12 months Ongoing symptoms despite conservative measures FI episodes > 1x/week
Exclusion criteria	Malignant disease Rectal bleeding of unknown origin Inflammatory bowel disease	Malignancy Inflammatory bowel disease Untreated rectal prolapse Acute perianal sepsis Obstructed defaecation syndrome or chronic constipation Neurological disease Previous rectal resection and sphincter defects > 120º
Clinical outcome measures	Clinical effectiveness: FI severity (Vaizey [= St Mark’s incontinence score]) Safety: migration of prostheses (3D endo-anal ultrasound)	Clinical effectiveness: FI severity (CCFI, Vaizey), QoL (FIQL) Safety: positioning of the prostheses (3D endo-anal ultrasound)
FU, months	Median: 8 (range 3–18)	3, 12
Outcomes		
Clinical effectiveness		
Faecal incontinence severity		
Vaizey (points [36] or mean ± SD [37])		
Preoperative	22 points	10.5 ± 9.5
Postoperative 3 months	NR	9.0 ± 10.8
Postoperative 8 months	13 points; *p* = 0.008	NR
Postoperative 12 months	NR	9.0 ± 10.3; *p* = 0.264
CCFIS (mean ± SD [37])		
Preoperative	NR	10.8 ± 4.9
Postoperative 3 months	NR	9.3 ± 5.8
Postoperative 12 months	NR	8.3 ± 6.2; *p* = 0.175
FIQL: lifestyle (mean ± SD [37])		
Preoperative	NR	2.8 ± 2.8
Postoperative 3 months	NR	3 ± 1.5
Postoperative 12 months	NR	3.4 ± 1.7; *p* = 0.527
FIQL: coping/behaviour (mean ± SD [37])		
Preoperative	NR	1.9 ± 0.9
Postoperative 3 months	NR	2.4 ± 1.0
Postoperative 12 months	NR	2.6 ± 1.0; *p* = 0.047
FIQL: depression/self-perception (mean ± SD [37])		
Preoperative	NR	2.75 ± 1
Postoperative 3 months	NR	3.3 ± 1.6
Postoperative 12 months	NR	3.1 ± 1.5; *p* = 0.132
FIQL: embarrassment (mean ± SD [37])		
Preoperative	NR	2.2 ± 1.0
Postoperative 3 months	NR	2.3 ± 0.8
Postoperative 12 months	NR	2.6 ± 1.0; *p* = 0.156
Outcomes		
Safety (n [%])		
Procedure-related adverse events		
Intraoperative complications	0 (0)	1 (7.7) (rectal perforation)
Postoperative complications/morbidity	NR	NR
Infection/sepsis/ inflammation	NR	NR
Anal discomfort/pain, analgesia > 48 h	1 (9)	NR
Adverse effect/reaction/general complication	NR	NR
Device-related adverse events		
Dislodgement of prostheses	10 (91)	NR
Prosthesis removed/extruded	1 (9)	3 (23.1)

*CCFIS* Cleveland Clinic Faecal Incontinence score, *CoI* conflict of interest, *FI* faecal incontinence, *FIQL* Faecal Incontinence Quality of Life score, *FU* follow-up, *NR* not reported, *pts* patients, *QoL* quality of life, *SD* standard deviation, *yrs* years

Scores:

CCFIS: The *Wexner Cleveland Clinic Faecal Incontinence Score (CCFIS)* includes five parameters regarding the type of incontinence and five response options [30]. The total score ranges from 0 (normal continence) to 20 (total incontinence) [30]

Vaizey: The *Vaizey score* is similar to the CCFIS [31]. The total score ranges from 0 (perfect continence) to 24 (totally incontinent) [31]

FIQL: The *Faecal Incontinence Quality of Life Scale (FIQL)* comprises 29 items and forms four subscales, including lifestyle, coping/behaviour, depression/self-perception, and embarrassment [24, 32]. The scale ranges from 1 (low status of QoL) to 5 (high status of QoL) [32]

AMS: The *American Medical Systems score (AMS)* is a modification of the FIQL and assesses the physical, psychological and social impact, pad use, lifestyle alterations, embarrassment/shame, depression, and coping/behaviour [24]. The AMS score ranges from 0 (high status of QoL) to 120 (low status of QoL) [10]

### Quality appraisal

Extracted data were independently assessed (LG, CW) for internal validity and risk of bias (RoB) using the Institute of Health Economics (IHE) RoB checklist for case series (Additional file 1: Table A-1) [33]. Disagreements were resolved through discussion or involving a third author (MW). The overall RoB was assessed using a predefined point score (range: 0–20; low RoB: > 18, moderate RoB: 14.5–18, high RoB: ≤ 14). Therefore the answers to the specific questions of the IHE checklist were added up with no: 0, partial/unclear: 0.5, and yes: 1 point.

The strength of the available evidence was assessed across the outcomes according to the Grading of Recommendations Assessment, Development and Evaluation (GRADE) approach [34] (Table 4). Each outcome was individually judged according to study design, RoB, inconsistency, indirectness, imprecision, and other considerations.

**Table 4:** GRADE evidence profile: Clinical effectiveness and safety of implantable bulking agents in patients with faecal incontinence

Quality assessment								
Number of studies (patients)	Study design	Risk of bias	Inconsistency	Indirectness	Imprecision	Other considerations	Impact	Certainty (Importance)
Clinical effectiveness								
Due to the lack of a controlled group, no data on clinical effectiveness outcomes can be compared and synthesised								
Safety								
Procedure-related adverse events (FU: range 1 month to 36 months)								
8 (166 pts)	Single-arm, before-after study	Very serious^a^	Not serious	Not serious	Not serious	None	In 16 of 166 analysed pts Intraoperative complications: n = 4 Postoperative complications/morbidity: n = 0 Infection/sepsis/inflammation: n = 0 Anal discomfort/pain, analgesia > 48 h: n = 12 Adverse effect/reaction/general complication: n = 0	⊕◯◯◯ VERY LOW (crucial)
Device-related adverse events (FU: range 1 month to 36 months)								
8 (166 pts)	Single-arm, before-after study	Very serious^a^	Not serious	Not serious	Not serious	None	In 48 of 166 analysed pts Dislodgement of prostheses: n = 41 Prosthesis removed/extruded: n = 7	⊕◯◯◯ VERY LOW (crucial)

*FU* follow-up, *GRADE* Grading of Recommendations, Assessment, Development and Evaluations, *pts* patients

Nomenclature for GRADE table:

Limitations: 0: no limitations or no serious limitations; − 1: serious limitations

Inconsistency: NA: Not applicable (only one trial); 0: no important inconsistency; − 1: important inconsistency

Indirectness: 0: direct, no uncertainty, − 1: some uncertainty, − 2 major uncertainty

Other modifying factors: publication bias likely (− 1), imprecise data (− 1), strong or very strong association (+ 1 or + 2), dose–response gradient (+ 1), Plausible confounding (+ 1)

^a^Using the IHE risk of bias checklist, three studies were rated with moderate and three studies with a high risk of bias (Additional file 1). Very serious limitations are given due to the lack of controlled study designs

### Data synthesis

A qualitative synthesis of the evidence was used to analyse the data. No further statistical analyses were performed.

## Results

### Search results

The database search resulted in 159 records after deduplication (see PRISMA diagram, Fig. 2). During the abstract screening, 128 references were excluded, resulting in 31 full-text articles assessed for eligibility. A further 25 records were excluded during full-text screening because they did not meet the inclusion criteria, resulting in six articles eligible for the evidence synthesis. The update search revealed 47 references, whereof two studies were included according to the inclusion criteria. Therefore, in total eight studies could be included in the analysis. 

### Characteristics of included studies

Tables 2 and 3 provide an overview of the study characteristics and data extraction. No comparative trials could be identified. The evidence consists of eight prospective single-arm, before-after studies fulfilling the inclusion criteria for assessing clinical effectiveness and safety of implantable bulking agents. Of those, seven studies were single-centred [7, 12, 17, 26, 35], and one trial was conducted at multiple centres [10]. All studies, except three (Spain [12], Austria [36], Australia [37]), were conducted in Italy. They were carried out between 2011 [10] and 2022 [7, 26, 37]. The sponsor was not reported [7, 10, 12, 17, 36, 37], or it was declared that there was no commercial sponsor.

Among these eight studies, 173 patients were enrolled, and 166 of them were analysed. Losses to follow-up (FU) were reported in two studies (n = 7) [26, 37]. Eighty-one patients received Gatekeeper™ implants, and 85 patients received Sphinkeeper™ prostheses. The individual patients were treated with four to six Gatekeeper™ prostheses [10, 12, 35] or nine to ten Sphinkeeper™ prostheses [7, 17, 26]. The age of patients ranged from 20 [17] to 89 [36] years. The assessed indications were passive FI [12, 35], passive, urge, or mixed FI [17], refractory FI [36], and four trials did not specify the form of FI [7, 10, 26, 37]. All studies, except two [7, 17], analysed short-term effectiveness *and* sustainability for more than six months, i.e., durability of implantable bulking agents' effects. One study only assessed sustainability (8 months FU) [36]. The number of patients per study at baseline ranged from seven [12] to 54 [10], and the FU period ranged from one [10, 12, 26, 35] to 36 [35] months.

A search in three clinical trials registries identified ongoing and unpublished studies resulting in 13 trials, whereof four relevant studies could be found. Three studies could be identified during the update search in the clinical trials registries.

### Risk of bias assessment

Across the eight included studies, the overall RoB was moderate (n = 5) [10, 26, 35–37] or high (n = 3) [7, 12, 17]. The main reasons for bias were the single-centre study designs, lack of patient characteristics, different patients' point of disease (FI onset/duration) when entering the study, and non-blinded outcome assessors. Further reasons were unclear consecutive recruitment, not stated exclusion criteria, not described co-interventions, not established outcome measures a priori, no information about statistical tests and quantitative data, short FUs, results did not support conclusion, or not reported competing interests and sources of support.

### Clinical effectiveness

In the absence of data from controlled trials, no comparisons could be made between implantable and injectable bulking agents for FI's treatment. The outcome FI severity was assessed in 166 patients, QoL in 96 patients, and safety outcomes in 166 patients. In the present review, FUs at three months and the last FUs (i.e., six, eight, 12, 14, or 36 months) after surgery were compared.

#### FI severity

FI severity was assessed by the instruments CCFIS and/or the Vaizey Score. Seven studies (155 patients) measured FI severity with the *CCFIS*. In five of these seven studies [7, 10, 12, 26, 35] (133 patients) the CCFIS improved, whereas in one study no statistically significant improvements could be observed [37], and one study [17] did not report postoperative data. FI severity statistically significantly improved from baseline (mean ± SD) 12.4 ± 1.8 to 3-months FU 4.9 ± 1.5 (*p* < 0.0001) and 36-months FU 4.9 ± 1.7 (*p* < 0.0001; 20 patients) in one study [35]. In another study, CCFIS improved from preoperative (mean ± SD) 16.0 ± 4.0 to 3-months FU 10.4 ± 3.2 (*p* < 0.01) and 12-months FU 10.1 ± 3.1 (*p* < 0.01; 7 patients) [12]. Six months after operation, FI severity improved (mean [range]) to 8.91 (6.0–12.0; *p* < 0.05) compared to baseline (12.46 [10.0–15.0]; 13 patients) [7]. After 12 months postoperative, FI severity improved from (median [range]) preoperative 12.0 (3.0–20.0) to 5.0 (0.0–16.0; *p* < 0.001; 54 patients) [10]. After 14 months, improvements from (median [1. and 3. quartiles]) 12.0 (9.0–15.0) to 7.0 (5.0–11.0; *p* < 0.01) could be observed (39 patients) [26].

The *Vaizey score* improved in three (104 patients) [10, 26, 36] of five studies. One study reported statistically non-significant improvements [37], and another study did not report postoperative data [17]. FI severity improved from (median [range]) 14.0 (3.0–24.0) to 6.5 (0.0–17.0; *p* < 0.001) at 12-months FU (54 patients) [10]. Furthermore, an improvement from (median [1. and 3. quartile]) 15.0 (13.0–18.0) to 14-months FU 11.0 (7.0–14.0; *p* < 0.01) was reported (39 patients) [26]. In the third study, an improvement from 22 to 13 points (*p* = 0.008) was found after eight months post surgery [36].

#### Disease-related quality of life

Five studies measured QoL with the *FIQL* (96 patients) [7, 10, 12, 17, 37]. Improved QoL could be found in one trial assessing lifestyle (*p* < 0.05), coping/behaviour (*p* < 0.01), depression/self-perception (*p* < 0.05), and embarrassment (*p* < 0.01) 12 months after surgery (54 patients) [10]. In another study [37], only the domain ‘coping/behaviour’ of the FIQL statistically significantly improved (*p* < 0.05; 12 patients). In two studies, QoL did not statistically significantly improve (20 patients) [7, 12]; one study (10 patients) [17] did not report the differences.

The *AMS* was additionally used in two studies (64 patients) [10, 17]. Here, QoL statistically significantly improved after 12 months from (median [range]) 87.0 (27.0–120.0) to 43.5 (0.0–106.0; *p* < 0.001) (54 patients) [10]. The second trial did not report any postoperative data [17].

### Patient safety

In total, 64 safety events occurred in the 166 patients analysed.

#### Procedure-related adverse events

Intraoperative complications were reported in six (including 136 patients) [7, 10, 12, 26, 36, 37] of eight studies and occurred in four analysed patients. Thereof, in three patients, prostheses were extruded during surgery [10], and one patient sustained an intraoperative rectal injury [37].

Postoperative complications, morbidity, infection, sepsis and inflammation, were reported in five studies [7, 10, 12, 17, 26] but did not occur in any of these trials. Anal discomfort, pain, and analgesia > 48 h were reported in six studies [7, 10, 12, 17, 26, 36] and occurred in twelve of 134 analysed patients [10, 12, 17, 26, 36]. Adverse effects, reactions and general complications were reported in three studies [17, 26, 35] but did not occur in any patient.

#### Device-related adverse events

Prostheses’ dislodgement, i.e., migration/dislocation, was reported in seven studies and occurred in 41 of 154 analysed patients [7, 10, 12, 17, 26, 35, 36]: Four (20%) [35], five (71%) [12], three (6%) [10], 18 (46%) [26], one (8%) [7], and ten (91%) [36] patients.Prostheses had to be removed or extruded in seven of 53 patients [7, 12, 36, 37], reported in five studies [7, 12, 17, 36, 37].

### Quality of evidence

According to GRADE schema [34], the strength of evidence was rated for safety outcomes only (Table 4). Strength of evidence on clinical effectiveness outcomes of implantable compared to injectable bulking agents could not be assessed due to the lack of controlled trials. The overall strength of evidence for implantable bulking agents' safety outcomes was rated *very low* due to the uncontrolled study design and very serious RoB.

## Discussion

This systematic review aims to assess implantable bulking agents' clinical effectiveness and safety, a minimal invasive second-line therapy after failure of conservative interventions. After conservative measures fail, bulking agents might be the final minimally invasive option in FI management [16]. Since implantable bulking agents (Gatekeeper™, Sphinkeeper™) are relatively new techniques, this report is—to our knowledge—the first systematic review based on the best available evidence. The systematic literature search identified eight prospective, before-after, single-arm studies. This limitation of quality of evidence entails that all included studies are highly prone to bias due to their uncontrolled before-after study design. The number of patients at baseline ranged between seven and 54 patients. Due to this high variability, findings have to be interpreted with caution. Among the eight examined studies, five are from Italy, which may be because an Italian company manufactures the devices. Three [10, 17, 26] of the eight included studies were conducted at the same institution. This may be a result as similar study teams conducted the trials.

The main finding is that FI severity (CCFIS and Vaizey Score) statistically significantly improved in six of eight studies [7, 10, 12, 26, 35, 36]. Clinically relevant improvement of FI severity compared to baseline was denoted with a minimum of a 50% reduction in severity scales and number of FI episodes [12]. In this review, clinically relevant improvements in FI severity could be observed after three [12], six [7], 12 [12], and 14 [26] months. Regarding the number of FI episodes, the clinically relevant improvement could be shown in one study after three and 12 months [12]. It must be mentioned that the only multicentred study describes some of the best clinical and functional outcomes included in the review where Gatekeeper™ was used [10]. Furthermore, Leo et al. had to be excluded due to its retrospective design [1]. This trial included 27 patients who underwent Shinkeeper™ surgeries. No intra-operative complications were reported. The Vaizey score significantly improved from baseline testing (*p* < 0.00016), and half of the patients achieved a 50% reduction in the score [1].

Furthermore, non-crucial outcomes were the number of FI episodes, soiling, gas, liquid and solid stool. The number of FI episodes (per week [7] or per month [12]) was measured in two studies and statistically significantly improved in both trials after three [12], six [7], and 12 months [12]. Soiling, gas, liquid stool, and solid stool were reported in three studies, whereof one study [17] did not report postoperative data. Liquid and solid stool statistically significantly improved after three, 12 [10], and 14 [26] months. Soiling and gas statistically significantly improved three, 12 [10] (patients with ≥ 75% improvement in FI) and 14 [26] months after implantation.

Other patient-relevant outcomes, such as deferment of defaecation or subanalyses (e.g., influence of obstetric trauma), were not subject to the present review. FI is also defined as the inability to defer defaecation and evacuation to socially convenient times [35]. One year after Gatekeeper™ implantation, 80% of patients could defer defaecation for at least five minutes [10]. Further publications affirm improved deferment for a minimum of five minutes after Sphinkeeper™ surgeries [2, 38–40].

The principal aetiologic factor for FI in females is obstetric trauma [5]. Functional and/or structural abnormalities of the EAS and IAS are often secondary to traumatic vaginal delivery and, therefore, more common in women [5]. Many females had anal sphincter defects or lesion due to obstetric trauma or injuries at baseline (5/10 [16]; 9/15 [41]; 14/36 [42]; 13/14 [43]; 10/18 [1]). Unfortunately, no subgroup analyses were presented. However, implantable bulking agents can be effective in the presence of a history of obstetric anal sphincter injury [41].

Astoundingly, in only one of five studies, QoL improved statistically significantly [10]. It is expected that FI impacts QoL as patients are unable to control stool/flatus, leading to embarrassment, fear of such FI episodes, and limitations in daily life and activities [32]. QoL instruments should not be considered as a direct indicator of FI severity because the same (objective) severity level can affect different patients in a dissimilar way [44] and the validation of the FIQL was only through translations [45]. Nonetheless, the FIQL has met psychometric criteria for validity and reliability and is recommended for assessing QoL in FI patients [32, 45]. Patients' QoL is crucial; thus, it must be underlined that it is challenging at this point in time lacking high-quality evidence to assess the effectiveness.

Dislodgement of prostheses occurred in 41 of 154 analysed patients. Prosthetic displacement is a common adverse event with rates ranging between 14 and 71%, measured in a retrospective cohort analysis using three-dimensional endoanal ultrasound [16]. The main cause of a possible progressive decline in a therapeutic effect are displacements of bulking agents [12]. Nonetheless, prosthetic displacements negatively correlate with postoperative changes in FI severity measured by CCFIS after 12 months [16]. Furthermore, implantable bulking agents can be replaced after removing protruded prostheses [16].

### Gatekeeper™ vs Sphinkeeper™

A comparison between the two products was conducted in a small age-matched cohort study (n = 20) [16]. The superiority of using a higher number and greater size of Sphinkeeper™ prostheses was shown in this comparative analysis regarding FI severity and muscle tension [16]. Furthermore, in a subgroup analysis (four vs six Gatekeeper™ prostheses in 20 patients), better results in terms of FI severity after implanting six prostheses were shown [35]. To conclude, more prostheses might be more effective [3], and Sphinkeeper™ might be indicated in patients with a more severe sphincter malfunction [46]. As implantable bulking agents, i.e. Gatekeeper™ and Sphinkeeper™, are manufactured by the same company (THD s.p.A., Italy), a lack of comparators exist. The comparison of Gatekeeper™ and Sphinkeeper™ is based on data produced by other analyses as our research question did not address this comparison.

### Limitations

The main limitation of the present review is that only prospective studies were taken into account to reduce possible confounders. Nevertheless, retrospective studies might have provided additional information on safety and contextual aspects. Furthermore, we did not consider delay defecation in our report and comparing Gatekeeper™ and Sphinkeeper™ was not the focus of our report. Above all, the key limitation of the included evidence is that all identified studies are highly prone to bias due to their uncontrolled before-after study design. Due to the lack of comparative studies, no information on the relative clinical effectiveness compared to injectable bulking agents can be given. This limit on available studies and the strong need for comparative trials demand further studies. Nevertheless, currently, six relevant ongoing studies examine bulking agents. One randomised controlled trial (ISRCTN00247) compares anal bulking agents vs sacral nerve stimulation (n = 100) with > 50% reduction of the number of FI episodes as the primary outcome. Three observational studies (NCT03080753, NCT04664868, ISRCTN61603070) are conducted with small numbers of patients (n = 11–52) with different primary outcomes such as FI severity, postoperative infection, pain, psychological/physical well-being, migration of prostheses, and QoL.Two additional ongoing observational studies could be identified during the update search. One trial (NCT030807539) is currently conducted involving 52 patients with severity of anal incontinence as the primary outcome. The other trial (NCT05222217) involves 13 patients and measures changes in the number of gas incontinence and soiling episodes as primary outcomes.

The small number of included participants across the studies (7–54 patients) could have influenced (serious) adverse events' occurrence. Another major limitation is that all of the clinical outcomes were patient-reported, although the used questionnaires are validated. Only safety outcomes based on narrative descriptions could be captured and analysed within the GRADE scheme in the eight prospective studies' analyses. Furthermore, Ratto et al. [17] trial had a very short FU, i.e., only three months, which may be too short for assessing FI patients. This systematic review excluded retrospective studies and possible safety data could have been missed.

## Conclusion

FI is a highly relevant topic, not only due to demographic changes but also because of its stigmatising impact on an individual's well-being. It is crucial to understand patients' FI symptoms and severity to direct each patient to the most effective treatment pathway. Fortunately, most FI patients profit from conservative measures and the importance of these treatments must be highlighted. In the absence of comparative data, it is impossible to ascertain the relative benefit and risk of implantable compared to injectable bulking agents.

Implantable bulking agents might be an effective and safe minimally invasive approach in FI treatment under restrictions until controlled trials are available; clinical implementation is only considered as second-line therapy if conservative therapies fail. Considering safety events, even if not serious, it must be noted that dislodgement of prostheses must be taken into account for clinical applications and decision making and need to be investigated in further studies. Minimally invasive implantable bulking agents of self-expanding prostheses in the anal sphincter were included in the 2022 version of the Austrian hospital benefit catalogue as preliminary code (XN170) for observation purposes.

The National Institute for Health and Care Excellence (NICE) also concluded in their interventional procedure guidance that evidence on safety and efficacy is inadequate in quality and quantity; this procedure should only be used in the context of research [47, 48] and under documentation. In the analysed studies, the severity of FI improved statistically significantly, but not so QoL. This discrepancy needs to be explored in further studies.

## Supplementary Information

**Additional file 1:** “Risk of bias of included studies (according to the Institute of Health Economics [IHE] checklist for case series): Implantable bulking agents for faecal incontinence”: This table presents the risk of bias of the eight included studies according to the Institute of Health Economics (IHE) checklist for case series.

## Data Availability

The datasets used and/or analysed during the current study are available from the corresponding author on reasonable request and from the published AIHTA report (https://eprints.aihta.at/1322/1/DSD_87_Update2021.pdf).

## Abbreviations

AMS: American Medical Systems score
CCFIS: Cleveland Clinic Faecal Incontinence Score
EAS: External anal sphincter
EU: European Union
EUnetHTA: European Network of Health Technology Assessment
FI: Faecal incontinence
FIQL: Faecal Incontinence Quality of Life Scale
FU: Follow-up
GRADE: Grading of Recommendations, Assessment, Development and Evaluation
IAS: Internal anal sphincter
IHE: Institute of Health Economics
NICE: National Institute for Health and Care Excellence
PICOS: Population, Intervention, Comparison, Outcome, and Study design
PRISMA: Preferred Reporting Items for Systematic Reviews and Meta-Analyses
QoL: Quality of life
RoB: Risk of bias
WHO: World Health Organisation
