# Supplementary material for: Clinical effectiveness and safety of self-expandable implantable bulking agents for faecal incontinence: a systematic review
Source: BMC Gastroenterol. 2022 Aug 17;22:389. doi: 10.1186/s12876-022-02441-4 (PMC9386976; doi:10.1186/s12876-022-02441-4)
Supplement: Supplementary file 1 — Additional file 1: “Risk of bias of included studies (according to the Institute of Health Economics [IHE] checklist for case series): Implantable bulking agents for faecal incontinence”: This table presents the risk of bias of the eight included studies according to the Institute of Health Economics (IHE) checklist for case series. [file 12876_2022_2441_MOESM1_ESM.docx]

## Literature search strategies

### Search strategy for Cochrane

| Search Name: Implantable Bulking Agents for Faecal Incontinence (MEL 2021) | |
| --- | --- |
| Last Saved: 17/12/2020 20:49:02 | |
| Comment: LG/CW | |
| ID | Search |
| #1 | MeSH descriptor: [Fecal Incontinence] explode all trees |
| #2 | ((faecal OR fecal OR anal OR bowel* OR gut* OR digesti* OR gastr*) NEAR (incontinen* or soil*)) (Word variations  have been searched) |
| #3 | MeSH descriptor: [Anal Canal] explode all trees |
| #4 | ("anal sphincter*") (Word variations have been searched) |
| #5 | #1 OR #2 OR #3 OR #4 (Word variations have been searched) |
| #6 | ("bulking agent*") (Word variations have been searched) |
| #7 | (Gatekeeper*) (Word variations have been searched) |
| #8 | (Gate-keeper*) (Word variations have been searched) |
| #9 | #6 OR #7 OR #8 (Word variations have been searched) |
| #10 | #5 AND #9 (Word variations have been searched) |
| #11 | (Sphinkeeper*) (Word variations have been searched) |
| #12 | #10 OR #11 (Word variations have been searched) |
| #13 | #12 with Publication Year from 2015 to 2020, in Trials (Word variations have been searched) |
| #14 | #12 with Cochrane Library publication date Between Jan 2015 and Dec 2020 (Word variations have been searched) |
| #15 | #13 OR #14 (Word variations have been searched) |
| Total hits: 31 | |

### Search strategy for Medline via Ovid

| Database: Ovid MEDLINE(R) and In-Process & Other Non-Indexed Citations and Daily <1946 to December 15, 2020>,  Ovid MEDLINE(R) and Epub Ahead of Print, In-Process & Other Non-Indexed Citations and Daily <2016 to December 15, 2020> | |
| --- | --- |
| Search date: 17.12.2020 | |
| ID | Search |
| 1 | exp Fecal Incontinence/ (11499) |
| 2 | ((faecal or fecal or anal or bowel* or gut* or digesti* or gastr*) adj3 (incontinen* or soil*)).mp. (17547) |
| 3 | exp Anal Canal/ (21113) |
| 4 | anal sphincter*.mp. (7317) |
| 5 | 1 or 2 or 3 or 4 (36160) |
| 6 | bulking agent*.mp. (1664) |
| 7 | Gatekeeper*.mp. (7301) |
| 8 | Gate-keeper*.mp. (494) |
| 9 | 6 or 7 or 8 (9426) |
| 10 | 5 and 9 (166) |
| 11 | Sphinkeeper*.mp. (10) |
| 12 | 10 or 11 (173) |
| 13 | limit 12 to yr="2015-2020" (85) |
| 14 | limit 13 to (english or german) (85) |
| 15 | remove duplicates from 14 (46) |

### Search strategy for CRD (DARE, NHS-EED, HTA)

| Search Name: Implantable Bulking Agents for Faecal Incontinence (MEL 2021) LG/CW | |
| --- | --- |
| Search date: 17.12.2020 | |
| ID | Search |
| 1 | MeSH DESCRIPTOR Fecal Incontinence EXPLODE ALL TREES |
| 2 | (faecal incontinen*) |
| 3 | (fecal incontinen*) |
| 4 | MeSH DESCRIPTOR Anal Canal EXPLODE ALL TREES |
| 5 | (anal sphincter* ) |
| 6 | ((faecal OR fecal OR anal OR bowel* OR gut* OR digesti* OR gastr*) NEAR (incontinen* or soil*)) |
| 7 | #1 OR #2 OR #3 OR #4 OR #5 OR #6 |
| 8 | (bulking agent*) |
| 9 | (Gate*) |
| 10 | #8 OR #9 |
| 11 | #7 AND #10 |
| 12 | (Sphinkeeper*) |
| 13 | #11 OR #12 |
| 14 | (#13) FROM 2015 TO 2020 |
| Total hits: 2 | |

### Search strategy for Embase

| Database Name: Embase.com | | |
| --- | --- | --- |
| Search date: 17.12.2020 | | |
| No. | Query Results | Results |
| #16. | #15 AND ([english]/lim OR [german]/lim) | 134 |
| #15. | #14 AND [2015-2020]/py | 135 |
| #14. | #12 OR #13 | 417 |
| #13. | sphinkeeper*:ti,ab,kw,de,lnk,dn | 17 |
| #12. | #6 AND #11 | 405 |
| #11. | #7 OR #8 OR #9 OR #10 | 107,383 |
| #10. | 'gate-keeper*':ti,ab,kw,de,lnk,dn | 591 |
| #9. | gatekeeper*:ti,ab,kw,de,lnk,dn | 7,157 |
| #8. | 'bulking agent*' | 2,639 |
| #7. | 'bulking agent'/exp | 99,298 |
| #6. | #1 OR #2 OR #3 OR #4 OR #5 | 34,481 |
| #5. | (faecal OR fecal OR anal OR bowel* OR gut* OR digesti* OR gastr*) NEAR/3 (incontinen* OR soil* OR sphincter*) | 23,625 |
| #4. | 'anus sphincter'/exp | 9,281 |
| #3. | 'fecal incontinence' | 8,228 |
| #2. | 'faecal incontinence' | 3,704 |
| #1. | 'feces incontinence'/exp | 21,619 |

### Search strategy for HTA-INAHTA

| Date of search: 17.12.2020 | |
| --- | --- |
| Nr. | Search query,"Hits","Searched At" |
| 12 | (Sphinkeeper*) OR (((Gate-keeper*) OR (Gatekeeper*) OR ("bulking agent*")) AND (("anal sphincter*") OR ("Anal Canal"[mhe]) OR ((faecal OR fecal OR anal OR bowel* OR gut* OR digesti* OR gastr*) AND (incontinen* OR soil*)) OR  ("Fecal Incontinence"[mhe]))),"1","2020-12-17T16:39:19.000000Z" |
| 11 | Sphinkeeper*,"0","2020-12-17T16:37:52.000000Z" |
| 10 | ((Gate-keeper*) OR (Gatekeeper*) OR ("bulking agent*")) AND (("anal sphincter*") OR ("Anal Canal"[mhe]) OR ((faecal OR fecal OR anal OR bowel* OR gut* OR digesti* OR gastr*) AND (incontinen* OR soil*)) OR ("Fecal Incontinence"[mhe])),"1","2020-12-17T16:37:24.000000Z" |
| 9 | (Gate-keeper*) OR (Gatekeeper*) OR ("bulking agent*"),"13","2020-12-17T16:37:14.000000Z" |
| 8 | Gate-keeper*,"5","2020-12-17T16:36:50.000000Z" |
| 7 | Gatekeeper*,"6","2020-12-17T16:36:33.000000Z" |
| 6 | "bulking agent*","2","2020-12-17T16:36:11.000000Z" |
| 5 | ("anal sphincter*") OR ("Anal Canal"[mhe]) OR ((faecal OR fecal OR anal OR bowel* OR gut* OR digesti* OR gastr*) AND (incontinen* OR soil*)) OR ("Fecal Incontinence"[mhe]),"67","2020-12-17T16:35:41.000000Z" |
| 4 | "anal sphincter*","19","2020-12-17T16:35:27.000000Z" |
| 3 | "Anal Canal"[mhe],"14","2020-12-17T16:35:03.000000Z" |
| 2 | (faecal OR fecal OR anal OR bowel* OR gut* OR digesti* OR gastr*) AND (incontinen* OR soil*),"57","2020-12-17T16:34:39.000000Z" |
| 1 | "Fecal Incontinence"[mhe],"21","2020-12-17T16:33:23.000000Z" |
| Total Hits: 0 Search query #12 limited to English/German | |

### Search strategy for clinical trial registries

**ClinicalTrials.gov** (Expert Search Mode)
Date of Search: 14.01.2021

AREA[ConditionSearch] ( Fecal Incontinence OR Faecal Incontinence OR Fecal Soiling OR Faecal Soiling OR Anal Incontinence OR Bowel Incontinence ) AND AREA[InterventionSearch] ( Bulking AND ( sphincter OR implant OR implantable ) OR Gatekeeper OR gate-keeper OR Sphinkeeper ) AND AREA[LastUpdatePostDate] EXPAND[Term] RANGE[01/01/2015, 01/14/2021]

**3** Studies identified

**WHO ICTRP** Basic Search Mode (Advanced Search not available on 14.01.2021)

*Population:* Fecal Incontinence, Faecal Incontinence, Anal Incontinence, Bowell Incontinence,
Fecal Soiling, Faecal Soiling

*Intervention:* Bulking (agent), Gatekeeper, Gate-keeper, Sphinkeeper

NB: Each of the “Population” terms was manually entered and combined (with the Boolean “AND” Operator) with each of the above -also manually entered- “Intervention” terms. The (“Intervention”) term ‘sphinkeeper’ was additionally searched individually without Boolean combinations.

12 (**10** additional) studies identified

**EU Clinical Trials** [EudraCT] (Basic Search Mode)
Date of Search: 14.01.2021

Fecal Incontinence OR Faecal Incontinence OR Fecal Soiling OR Faecal Soiling OR Anal Incontinence OR Bowel Incontinence) AND (Bulking OR Gatekeeper OR gate-keeper OR Sphinkeeper)

Date range: 01.01.2015-14.01.2021

No studies identified

## Risk of bias table

Table A‑1: Risk of bias of included studies (according to the Institute of Health Economics [IHE] checklist for case series): Implantable bulking agents for faecal incontinence.

| Study  reference/ID | Brusciano, 2020 [35] | De la Portilla, 2017 [12] | Ratto,  2016b [10] | Litta,  2021 [26] | La Torre,  2020 [7] | Ratto,  2016a [17] | Dawoud, 2021 [36] | Colbran, 2022 [37] |
| --- | --- | --- | --- | --- | --- | --- | --- | --- |
| Study objective | | | | | | |  |  |
| 1. Was the hypothesis/aim/objective of the study clearly stated? | Yes | Yes | Yes | Yes | Yes | Yes | Yes | Yes |
| Study design | | | | | | |  |  |
| 2. Was the study conducted prospectively? | Yes | Yes | Yes | Yes | Yes | Yes | Yes | Yes |
| 3. Were the cases collected in more than one centre? | No | No | Yes | No | No | No | No | No |
| 4. Were patients recruited consecutively? | Yes | Unclear | Yes | Yes | Yes | Yes | Yes | Unclear |
| Study population | | | | | | |  |  |
| 5. Were the characteristics of the patients included in the study described? | No | No | Yes | Yes | No | Yes | Yes | Yes |
| 6. Were the eligibility criteria (i.e., inclusion and exclusion criteria) for entry into the study clearly stated? | Yes | Partial^[[1]](#footnote-1)^ | Yes | Yes | Yes | Yes | Yes | Yes |
| 7. Did patients enter the study at a similar point in the disease? | Unclear^[[2]](#footnote-2)^ | No^[[3]](#footnote-3)^ | No^[[4]](#footnote-4)^ | No^[[5]](#footnote-5)^ | Unclear^[[6]](#footnote-6)^ | No^[[7]](#footnote-7)^ | No^[[8]](#footnote-8)^ | No^[[9]](#footnote-9)^ |
| Intervention and co-intervention | | | | | | |  |  |
| 8. Was the intervention of interest clearly described? | Yes | Yes | Yes | Yes | Yes | Yes | Yes | Yes |
| 9. Were additional interventions (co-interventions) clearly described? | No | No | Yes | Yes | Yes | Yes | No | No |
| Outcome measures | | | | | | |  |  |
| 10. Were relevant outcome measures established a priori? | Yes | Yes | Yes | Yes | Yes | No | Yes | No |
| 11. Were outcome assessors blinded to the intervention that patients received? | No | No | No | No | No | No | No | No |
| 12. Were the relevant outcomes measured using appropriate objective/subjective methods? | Yes | Yes | Yes | Yes | Yes | Yes | Yes | Yes |
| 13. Were the relevant outcome measures made before and after the intervention? | Yes | Yes | Yes | Yes | Yes | Yes | Yes | Yes |
| Statistical Analysis | | | | | | |  |  |
| 14. Were the statistical tests used to assess the relevant outcomes appropriate? | Yes | Unclear^[[10]](#footnote-10)^ | Yes | Yes | Yes | Unclear8 | Yes | Yes |
| Results and Conclusions | | | | | | |  |  |
| 15. Was follow-up long enough for important events and outcomes to occur? | Yes | Yes | Yes | Yes | No^[[11]](#footnote-11)^ | No^[[12]](#footnote-12)^ | Yes | Yes |
| 16. Were losses to follow-up reported? | Yes | Yes | Yes | Yes | Yes | Yes | Yes | Yes |
| 17. Did the study provided estimates of random variability in the data analysis of relevant outcomes? | Yes | Yes | Yes | Yes | Yes | Unclear^[[13]](#footnote-13)^ | Yes | Yes |
| 18. Were the adverse events reported? | Yes | Yes | Yes | Yes | Yes | Yes | Yes | Yes |
| 19. Were the conclusions of the study supported by results? | Yes | Yes | Yes | Yes | No^[[14]](#footnote-14)^ | Unclear8 | Yes | Yes |
| Competing interests and sources of support | | | | | | |  |  |
| 20. Were both competing interests and sources of support for the study reported? | Yes | No | Partial^[[15]](#footnote-15)^ | Yes^[[16]](#footnote-16)^ | No | Partial^[[17]](#footnote-17)^ | Yes | Yes |
| Points | 15.5 | 12 | 17.5 | 17 | 13.5 | 13 | 16 | 14.5 |
| Overall Risk of bias | Moderate | High | Moderate | Moderate | High | High | Moderate | Moderate |

1. Only inclusion criteria stated. [↑](#footnote-ref-1)
2. FI onset ≥6 months before the first visit. [↑](#footnote-ref-2)
3. Patients were treated “after having suffered from passive FI for a mean duration of 6 ± 2 years”. [↑](#footnote-ref-3)
4. Duration of FI (years) 3 (1–19); mean (range) [↑](#footnote-ref-4)
5. Duration of FI (years) 5 (2-10); median (first and third quartiles) [↑](#footnote-ref-5)
6. “FI (incontinence to liquid and/or solid stools) that had started at least 6 months before.” [↑](#footnote-ref-6)
7. Median duration of FI (years; range) 9 (3–21) [↑](#footnote-ref-7)
8. „The median duration of FI before surgery was 39 months (range 21–156).“ [↑](#footnote-ref-8)
9. „Eleven (84.7%) patients had suffered with FI for more than 3 years, with 15 (38.5%) having symptoms for over a decade.“ [↑](#footnote-ref-9)
10. No information given. [↑](#footnote-ref-10)
11. Last FU 6 months after surgery. [↑](#footnote-ref-11)
12. Last FU 3 months after surgery. [↑](#footnote-ref-12)
13. No quantitative data provided. [↑](#footnote-ref-13)
14. Significant results of the FIQL (Table 2) were missing and in the conclusion is reported about “promising results”. [↑](#footnote-ref-14)
15. Conflict of interest reported, but not sources of financial support. [↑](#footnote-ref-15)
16. The last author “received travel reimbursement by THD company to attend conferences and was proctor in courses on SphinKeeper implant”. [↑](#footnote-ref-16)
17. Source of financial support not provided. [↑](#footnote-ref-17)
